# Kinematics and muscle activity of individuals with incomplete spinal cord injury during treadmill stepping with and without manual assistance

**DOI:** 10.1186/1743-0003-4-32

**Published:** 2007-08-21

**Authors:** Antoinette Domingo, Gregory S Sawicki, Daniel P Ferris

**Affiliations:** 1 Division of Kinesiology, University of Michigan, Ann Arbor, MI, USA; 2 Department of Mechanical Engineering, University of Michigan, Ann Arbor, MI, USA; 3 Department of Biomedical Engineering, University of Michigan, Ann Arbor, MI, USA; 4 Department of Physical Medicine and Rehabilitation, University of Michigan, Ann Arbor, MI, USA

## Abstract

**Background:**

Treadmill training with bodyweight support and manual assistance improves walking ability of patients with neurological injury. The purpose of this study was to determine how manual assistance changes muscle activation and kinematic patterns during treadmill training in individuals with incomplete spinal cord injury.

**Methods:**

We tested six volunteers with incomplete spinal cord injury and six volunteers with intact nervous systems. Subjects with spinal cord injury walked on a treadmill at six speeds (0.18–1.07 m/s) with body weight support with and without manual assistance. Healthy subjects walked at the same speeds only with body weight support. We measured electromyographic (EMG) and kinematics in the lower extremities and calculated EMG root mean square (RMS) amplitudes and joint excursions. We performed cross-correlation analyses to compare EMG and kinematic profiles.

**Results:**

Normalized muscle activation amplitudes and profiles in subjects with spinal cord injury were similar for stepping with and without manual assistance (ANOVA, p > 0.05). Muscle activation amplitudes increased with increasing speed (ANOVA, p < 0.05). When comparing spinal cord injury subject EMG data to control subject EMG data, neither the condition with manual assistance nor the condition without manual assistance showed a greater similarity to the control subject data, except for vastus lateralis. The shape and timing of EMG patterns in subjects with spinal cord injury became less similar to controls at faster speeds, especially when walking without manual assistance (ANOVA, p < 0.05). There were no consistent changes in kinematic profiles across spinal cord injury subjects when they were given manual assistance. Knee joint excursion was ~5 degrees greater with manual assistance during swing (ANOVA, p < 0.05). Hip and ankle joint excursions were both ~3 degrees lower with manual assistance during stance (ANOVA, p < 0.05).

**Conclusion:**

Providing manual assistance does not lower EMG amplitudes or alter muscle activation profiles in relatively higher functioning spinal cord injury subjects. One advantage of manual assistance is that it allows spinal cord injury subjects to walk at faster speeds than they could without assistance. Concerns that manual assistance will promote passivity in subjects are unsupported by our findings.

## Background

Several investigators have shown that body weight supported treadmill training can improve walking ability in those with incomplete spinal cord injury [see Additional file 1] [1-8]. During this treatment, the patient is suspended in a standing position above a treadmill by means of a modified parachute harness so that the patient only bears a portion of his weight on their legs. A therapist on each side of the person then manually assists his legs through walking motions while the treadmill belt is moving. A third therapist may also stand behind the patient to help stabilize the trunk. One study showed that 80% of people with incomplete spinal cord injury who used a wheelchair for mobility became functional ambulators after body weight supported treadmill training [3]. The effects of this training were maintained long after the intensive treadmill training ended. However, Dobkin et al. performed a multi-center randomized clinical trial that had more equivocal results [7]. They found that body weight supported treadmill training was no more effective than highly intensive "conventional" physical therapy in improving walking ability. Clearly more research is needed to examine mechanisms and ideal training parameters for body weight supported treadmill training.

Recently, Hidler highlighted the need for more evidence supporting the choice of specific training parameters [9]. The amount of body weight support and the walking speed are just a few of the parameters that can greatly vary during treatment. We do not know what is the most effective and efficient manner to set these parameters or how to progress them as a patient makes functional gains. Another factor of training to consider is the use of functional electrical stimulation with locomotor training. Several studies have found therapeutic effects of functional electrical stimulation during gait rehabilitation [10-12], but like body weight support and walking speed, it is not clear how to optimize its use.

Another parameter of body weight supported treadmill training that needs to be considered is the amount of mechanical assistance that should be given and the manner in which it is given. One approach is to allow patients to practice stepping on a treadmill with body weight support but no mechanical assistance at all. This could only be done for patients with sufficient motor ability so that body weight support alone facilitated stepping. When this is not possible, the most readily available and most used form of assistance is manual. Unfortunately, this is also very labor intensive and requires a high level of skill to administer. The assistance given could vary from step to step and/or from trainer to trainer. To address these limitations, several groups have developed robotic devices to provide mechanical assistance during stepping [13-17].

One possible downside to manual or robotic assistance during body weight supported treadmill training is diminished motor learning. Physical guidance improves performance during the learning phase of an upper limb task while guidance is given, but the improvement in performance is not retained once the guidance is removed [18-20]. There is no clear evidence on how guidance affects learning in cyclical lower limb tasks. A fundamental assumption of body weight supported treadmill training is that it promotes activity dependent plasticity to improve function ability. Activity dependent plasticity depends on sufficient and appropriate voluntary drive to promote modifications in synaptic connections [21,22]. If manual assistance promotes passivity, then it may be detrimental because diminished neural activation limits the possibility of neural plasticity in relevant circuits.

In contrast, physical guidance may be necessary to learn how to perform a walking movement correctly. Presumably, manual assistance during body weight supported treadmill training helps to ensure that the patient is experiencing the correct kinematics of walking. This could be important because sensory information is an input to the locomotor neural networks. Afferent feedback directly influences the spinal generation of muscle activity that produces human walking [23-28]. Therefore, manual assistance could result in afferent feedback more typical of non-disabled persons during stepping practice. In addition, there are some situations in which learning a movement without physical guidance could be dangerous. When learning to walk after spinal cord injury, manual assistance certainly increases safety, especially when walking at faster speeds.

The purpose of this study was to determine how manual assistance affects lower limb electromyographic (EMG) activity and joint kinematics in subjects with incomplete spinal cord injury during body weight supported treadmill training. There are two competing hypotheses on how EMG activity might be affected by treadmill training with manual assistance. One possibility is that manual assistance decreases the patient's effort, thereby reducing EMG amplitudes. An alternative possibility is that manual assistance provides more normative kinematic patterns, resulting in more appropriate sensory feedback and increasing EMG amplitudes. We examined individuals with incomplete spinal cord injury that were able to walk with and without manual assistance at multiple speeds during body weight supported treadmill training to compare kinematics and muscle activation. The findings of this study should help to determine if manual assistance affects EMG activity and joint excursions for body weight supported treadmill training.

## Methods

### Subjects

We tested six adult volunteers with an incomplete spinal cord injury and six neurologically intact adult volunteers. Six subjects with incomplete spinal cord injury (ASIA Impairment Scale Classification of C or D) at the cervical or thoracic level participated in the study. Subjects were at least 12 months post-injury and free of any conditions that would limit their ability to safely complete testing. Five of six subjects were community ambulators with preferred over ground walking speeds of 0.37–0.95 m/s. Of these five subjects, four used canes. Table 1 details the cause, classification, level of spinal injury, preferred walking speed, and assistive devices of each subject. Six control subjects (age = 25.8 ± 2.9 years, mass = 66.7 ± 13.4 kg, mean ± SD) without neurological injury also participated in the study. The University of Michigan Institutional Review Board approved this project and all subjects gave informed consent prior to participating.

**Table 1:** Subject Information. Data for each subject showing age, body size, injury level, walking ability, body weight support level and walking speeds completed during the study.

**Subject**	**Age (yrs.)**	**Sex** **Height (cm)** **Weight (kg)**	**Injury** **Etiology**	**Injury Level**	ASIA* Level	**Post Injury (mos.)**	**Walking Aids**	**Overground Walking Speed (m/s)**	**BWS Level (%)** **Speeds w/o MA (m/s)** **Speeds w/MA (m/s)**
A	54	F	Dermoid	T11/T12	C	64	Cane (L, R)	0.41	30%
		165.1 cm	Tumor				Ankle-foot		0.18–0.89
		73.7 kg					orthosis (L)		0.18–0.89
B	52	F	Myxopapillary	T8/L2	D	93	Quad Cane (R)	0.61	30%
		156.2	Ependymoma						0.18–0.36
		58.1 kg							0.18–0.72
C	38	F	Transverse	T5	D	77	Cane (R)	0.37	50%
		175.3 cm	Myelitis				Ankle-foot		0.18–1.07
		115.3 kg					orthosis (L)		0.18–1.07
D	24	M	Trauma	T10/T11	D	111	-	0.95	30%
		185.4 cm							0.18–1.07
		101.5 kg							0.18–1.07
E	55	M	Sarcoidosis	C5/C6	C	144	Cane (R)	0.48	60%
		171.5 cm							0.18–0.54
		83.0 kg							0.18–0.89
F	50	M	Trauma	C4/C5	C	83	Wheelchair	-	50%
		193.0 cm					Soft ankle		0.18–0.72
		95.3 kg					brace (L, R)		0.18–1.07

* ASIA = American Spinal Injury Association Impairment Scale. A = Complete, E = Normal.

### Procedures

Subjects with spinal cord injury walked on a treadmill with and without manual assistance at six different speeds (0.18, 0.36, 0.54, 0.72, 0.89, 1.07 m/s) with body weight support (Robomedica, Inc., Irvine, CA). Additional video files show procedures at one speed for one subject [see Additional files 1 &2]. All subjects with spinal cord injury underwent one to two training sessions on the treadmill with body weight support prior to data collection to familiarize them with the procedure. The amount of body weight support and stepping speeds achieved varied between subjects due to their different walking abilities. Subjects with spinal cord injury were supported with 30% body weight support unless they required greater support to walk at multiple treadmill speeds. Initially, subjects were asked to walk with 30% body weight support without manual assistance. If they were unable to take steps at this level of support at 0.36 m/s, body weight support was increased in 10% increments until the subject could walk safely at this speed without manual assistance. Three subjects walked with 30% body weight support, two subjects walked with 50% body weight support, and one subject walked with 60% body weight support. The goal of the manual assistance was to minimize gait deviations (e.g., increasing step length, increasing toe clearance and hip flexion during swing). We attempted to collect data at all speeds for all subjects but only two subjects were able to walk at all six speeds with and without assistance. We collected data on the remaining subjects from the trials they were able to safely complete. Table 1 shows the stepping speeds each subject was able to achieve. Subjects who normally used lower limb orthoses wore them during testing to ensure their safety (Table 1). Control subjects walked on the treadmill without manual assistance at all speeds with 30% body weight support to match the baseline condition of the subjects with spinal cord injury.

The same trainers manually assisted all subjects following the procedures described by Behrman and Harkema for locomotor training with partial body weight support [6]. The trainers were instructed and supervised by a former trainer who was from the UCLA Human Locomotion Research Center that directed a large scale clinical trial on body weight supported treadmill training [29].

### Data acquisition and analysis

While walking under the two experimental conditions, we collected surface electromyographic and kinematic data. We used a Konigsberg Instruments, Inc. (Pasadena, CA) telemetry EMG system to record activity from eight muscles on one lower limb (tibialis anterior, TA; soleus, SO; medial gastrocnemius, MG; lateral gastrocnemius, LG; vastus lateralis, VL; vastus medialis, VM; rectus femoris, RF; and medial hamstring, MH). Inter-electrode distance was 2.5 cm for all subjects and muscles. Electrodes were circular with a diameter of 1.1 cm. We verified that cross-talk was negligible by visual inspection of the EMG signals[30]. We also used footswitches to delineate the stance phase and swing phase of gait. We placed electrogoniometers (Biometrics, Ltd., Ladysmith, VA) at the ankle, knee and hip joints on each leg to record joint angles. If the patient wore an ankle foot orthosis, the goniometer was placed on the outside of the orthosis. The computer collected all analog data at 1200 Hz for 15–25 seconds per trial depending on speed (Motion Analysis Corporation, Santa Rosa, CA). Subjects also wore footswitches as insoles to indicate the time each foot was or was not on the ground (B & L Engineering, Tustin, CA). Contacts in the footswitches were at the heel, fifth metatarsal, first metatarsal, and great toe to signify when those areas of the foot bearing weight. Subjects with spinal cord injury performed two trials of each condition (with and without manual assistance) and speed in a randomized order. Between 4 and 19 steps were analyzed per trial depending on speed. The difference in number of steps analyzed across trials and subjects was not likely to artificially alter the results [31]. Although some subjects could walk at faster speeds with manual assistance than they could without, only trials from speeds at which the subject could walk both with and without manual assistance were included. We only analyzed EMG and kinematic data from speeds that subjects could both walk with and without assistance because EMG amplitudes are a function of walking speed and including the data from the higher walking speeds would skew the results.

We used commercial software (Visual 3D, C-Motion, Inc., Rockville, MD) to process collected EMG and kinematic data. EMG data were high-pass filtered (20 Hz) to remove artifacts while preserving the integrity of the data, and then rectified and low-pass filtered (25 Hz). Kinematic data were low pass filtered at 6 Hz [32]. Averaged EMG and kinematic profiles were time normalized to the percentage of the stride cycle, beginning and ending with heel strike of the same foot. We calculated the EMG root-mean-square (RMS) for each step cycle within a trial for each muscle, and then averaged these values for an overall RMS value for each trial. We also calculated separate RMS values for the stance and swing phases of gait.

For each muscle, we normalized EMG RMS data to the highest average RMS that occurred in that muscle without manual assistance during one of the two trials at 0.36 m/s. We chose this speed for normalization because it was the highest speed that all subjects with spinal cord injury could achieve. Using JMP statistical software (Cary, NC), we used a repeated measure ANOVA (individual subject by speed by condition) to test for significant differences between normalized RMS values for the stance and swing phases separately. We also used a repeated measure ANOVA (individual subject by speed by condition) to test for significant differences between joint range of motion values. Tukey HSD post-hoc tests were performed to identify differences between specific groups. For power analyses, we calculated the least significant values, which gave the sensitivity of the test. We then compared the least significant values to the actual differences in group means to determine if testing any more subjects would likely change our results.

We performed cross-correlation analyses using Equation (1) to compare averaged electromyographic waveforms and kinematic profiles of control subjects with the profiles of each spinal cord injury subject with and without manual assistance [33-35].

R=Σxiyi(Σxi2)1/2(Σyi2)1/2,
 MathType@MTEF@5@5@+=feaafiart1ev1aaatCvAUfKttLearuWrP9MDH5MBPbIqV92AaeXatLxBI9gBaebbnrfifHhDYfgasaacH8akY=wiFfYdH8Gipec8Eeeu0xXdbba9frFj0=OqFfea0dXdd9vqai=hGuQ8kuc9pgc9s8qqaq=dirpe0xb9q8qiLsFr0=vr0=vr0dc8meaabaqaciaacaGaaeqabaqabeGadaaakeaacqWGsbGucqGH9aqpdaWcaaqaaiabfo6atjabdIha4naaBaaaleaacqWGPbqAaeqaaOGaemyEaK3aaSbaaSqaaiabdMgaPbqabaaakeaadaqadaqaaiabfo6atjabdIha4naaDaaaleaacqWGPbqAaeaacqaIYaGmaaaakiaawIcacaGLPaaadaahaaWcbeqaaiabigdaXiabc+caViabikdaYaaakmaabmaabaGaeu4OdmLaemyEaK3aa0baaSqaaiabdMgaPbqaaiabikdaYaaaaOGaayjkaiaawMcaamaaCaaaleqabaGaeGymaeJaei4la8IaeGOmaidaaaaakiabcYcaSaaa@4B7D@

where *x*_*i *_and *y*_*i *_are two series of data, and *i *= 0, 1, 2, ..., N-1. The first series of data was the averaged control subject data, and the second series was the data from individual subjects with spinal cord injury. Because the data were normalized to the percentage of the gait cycle, N = 101 in all analyses. We used the cross-correlation results to assess if manual assistance altered the shape and timing of muscle activation and kinematic profiles of subjects with spinal cord injury so that it was more similar to control subject profiles. We also performed cross-correlation analyses to compare EMG waveforms and kinematic profiles of subjects with spinal cord injury walking with manual assistance to walking without manual assistance. We performed repeated measure ANOVAs (individual subject by speed by condition) to test for significant differences in R-values and time lags. Tukey HSD post-hoc tests were performed to identify specific differences between groups. Power analyses were also carried out where appropriate.

We calculated coefficients of variation (CV) of EMG activation and joint angle profiles using Equation (2) to quantify variability of the different conditions [36].

CV=1N∑i=1Nσi21N∑i=1N|Xi|,
 MathType@MTEF@5@5@+=feaafiart1ev1aaatCvAUfKttLearuWrP9MDH5MBPbIqV92AaeXatLxBI9gBaebbnrfifHhDYfgasaacH8akY=wiFfYdH8Gipec8Eeeu0xXdbba9frFj0=OqFfea0dXdd9vqai=hGuQ8kuc9pgc9s8qqaq=dirpe0xb9q8qiLsFr0=vr0=vr0dc8meaabaqaciaacaGaaeqabaqabeGadaaakeaacqWGdbWqcqWGwbGvcqGH9aqpdaWcaaqaamaakaaabaWaaSaaaeaacqaIXaqmaeaacqWGobGtaaWaaabCaeaaiiGacqWFdpWCdaqhaaWcbaGaemyAaKgabaGaeGOmaidaaaqaaiabdMgaPjabg2da9iabigdaXaqaaiabd6eaobqdcqGHris5aaWcbeaaaOqaamaalaaabaGaeGymaedabaGaemOta4eaamaaqahabaWaaqWaaeaacqWGybawdaWgaaWcbaGaemyAaKgabeaaaOGaay5bSlaawIa7aaWcbaGaemyAaKMaeyypa0JaeGymaedabaGaemOta4eaniabggHiLdaaaOGaeiilaWcaaa@4CF4@

where *N *is the number of intervals over the stride, *X*_*i *_is the mean value of the variable at the *i*th interval, and *σ*_*i *_is the standard deviation of variable *X *about *X*_*i*_. We performed a repeated measure ANOVA (individual subject by speed by condition) to test for significant differences in the coefficients of variation of the joint angle profiles. We performed post-hoc tests and power analyses as described above.

## Results

Three of six subjects with spinal cord injury could walk at faster speeds with manual assistance than without. The average highest walking speed without manual assistance was 0.76 m/s. The average walking highest speed with manual assistance was 0.95 m/s (Table 1).

### Electromyography

**Figure 1:**
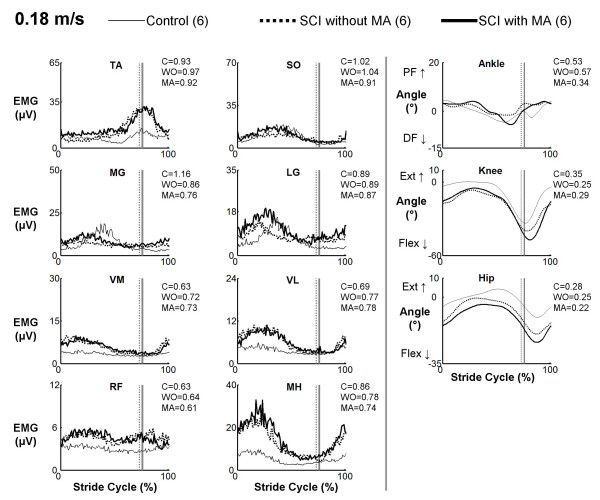
**EMG profiles for subjects with spinal cord injury walking with (MA) and without (WO) manual assistance and control (C) subjects at 0.18 m/s**. Averaged EMG profiles for tibialis anterior (TA), soleus (SO), medial gastrocnemius (MG), lateral gastrocnemius (LG), vastus medialis (VM), vastus lateralis (VL), rectus femoris (RF), and medial hamstring (MH) and averaged kinematic profiles for the ankle, hip and knee. Averages are taken from six subjects with spinal cord injury and six neurologically intact controls. Data from each subject were averaged over several step cycles within a trial, then over two trials of the same condition and speed, and finally averaged across subjects for the same condition and speed. Stride cycles were normalized from heel strike (0%) to heel strike of the same foot (100%). Vertical lines indicate the beginning of swing phase. The average coefficient of variation across subjects over the stride cycle is reported to the right of each plot.

**Figure 2:**
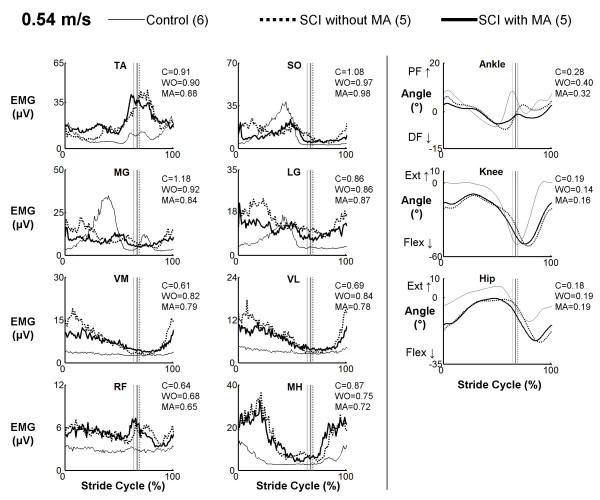
**EMG profiles for subjects with spinal cord injury walking with (MA) and without (WO) manual assistance and control (C) subjects at 0.54 m/s**. Averaged EMG profiles for tibialis anterior (TA), soleus (SO), medial gastrocnemius (MG), lateral gastrocnemius (LG), vastus medialis (VM), vastus lateralis (VL), rectus femoris (RF), and medial hamstring (MH) and averaged kinematic profiles for the ankle, hip and knee. Averages are taken from five subjects with spinal cord injury and six neurologically intact controls. Stride cycles were normalized from heel strike (0%) to heel strike of the same foot (100%). The average coefficient of variation across subjects over the stride cycle is reported to the right of each plot.

**Figure 3:**
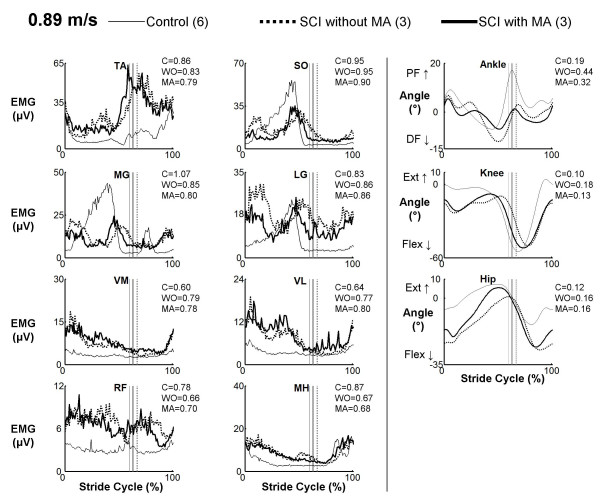
**EMG profiles for subjects with spinal cord injury walking with (MA) and without (WO) manual assistance and control (C) subjects at 0.89 m/s**. Averaged EMG profiles for tibialis anterior (TA), soleus (SO), medial gastrocnemius (MG), lateral gastrocnemius (LG), vastus medialis (VM), vastus lateralis (VL), rectus femoris (RF), and medial hamstring (MH) and averaged kinematic profiles for the ankle, hip and knee. Averages are taken from three subjects with spinal cord injury and six healthy controls. Stride cycles were normalized from heel strike (0%) to heel strike of the same foot (100%). The average coefficient of variation across subjects over the stride cycle is reported to the right of each plot.

There were clear differences between muscle activation patterns in subjects with spinal cord injury and control subjects. However, muscle activation profiles in subjects with spinal cord injury walking with manual assistance were very similar to profiles while walking without manual assistance (Figures 1, 2, and 3). Cross-correlation analyses of average EMG waveforms between with and without manual assistance produced correlation values greater than 0.89 and phase lags less than 2% (Table 2). When comparing spinal cord injury data to control data, neither the condition with manual assistance nor the condition without manual assistance showed a greater similarity to the control subject data (correlation and phase lag, ANOVA, p > 0.05). The exception was that when the subjects with SCI were given manual assistance, the profile of the vastus lateralis activation was more similar to the profile of the control subjects (p = 0.002, R = 0.91 without manual assistance, R = 0.93 with manual assistance). Power analyses showed that the differences in means of the R-values for TA, SO, LG, VM, and VL EMG profiles and the time shift for SO EMG profile were greater than the calculated least significant values. Therefore, this indicates that there is a 95% chance that there actually is no difference in R-values or time shift between the two conditions in these muscles [37].

**Table 2:** Cross-correlation analyses of EMG and kinematic profiles. Values shown are the results of cross correlation analyses comparing data for all speeds and conditions between: spinal cord injury subjects walking without manual assistance and control subject data (WO-Control), spinal cord injury subjects walking with manual assistance and control subject data (MA-control), and spinal cord injury subjects walking without manual assistance and with manual assistance (WO-MA). Waveforms and profiles were normalized to the percentage of the gait cycle and therefore the resulting shifts from the analyses are given in percentages. Statistical analyses were then performed (repeated measure ANOVAs) to find significant differences between R-values and time shifts.

		R-value	shift (%)			R-value	shift (%)
					
TA EMG	WO-Control	0.81	7	RF EMG	WO-Control	0.93	0
	MA-Control	0.82	5		MA-Control	0.93	0
	WO-MA	0.91*†	0*†		WO-MA	0.94	0
							
SO EMG	WO-Control	0.82	5	MH EMG	WO-Control	0.87	0
	MA-Control	0.84	2		MA-Control	0.86	0
	WO-MA	0.89*†	1		WO-MA	0.95*†	0
							
MG EMG	WO-Control	0.80	3	Ankle angle	WO-Control	0.47	-16
	MA-Control	0.80	2		MA-Control	0.37	-8
	WO-MA	0.90*†	0		WO-MA	0.77*†	2
							
LG EMG	WO-Control	0.83	3	Knee angle	WO-Control	0.87	-8
	MA-Control	0.85	-3		MA-Control	0.91*	-5*
	WO-MA	0.89*†	0		WO-MA	0.96*†	2*†
							
VM EMG	WO-Control	0.91	0	Hip angle	WO-Control	0.77	3
	MA-Control	0.92	0		MA-Control	0.78	4
	WO-MA	0.93*	0		WO-MA	0.92*†	1
							
VL EMG	WO-Control	0.91	0				
	MA-Control	0.93*	0				
	WO-MA	0.93	0				

*Indicates significantly different from WO-Control (p < 0.05)

† Indicates significantly different from MA-Control (p < 0.05)

**Figure 4:**
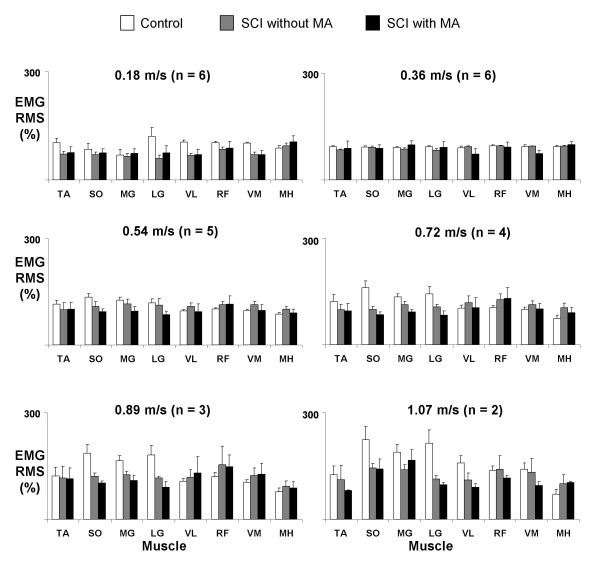
**Stance phase EMG RMS for subjects with spinal cord injury walking with and without manual assistance and control subjects at six different speeds**. Averaged normalized muscle activation amplitudes for tibialis anterior (TA), soleus (SO), medial gastrocnemius (MG), lateral gastrocnemius (LG), vastus medialis (VM), vastus lateralis (VL), rectus femoris (RF), and medial hamstring (MH) for the specified number of subjects with spinal cord injury and six control subjects. RMS data for each muscle were first normalized to the highest average RMS value that occurred among two trials at 0.36 m/s. These normalized values from each muscle were then averaged over two trials of the same condition and speed within a subject, and finally averaged across subjects for the same condition and speed. Bars indicate mean ± standard error. There were no significant differences in muscle activation amplitudes when walking with or without manual assistance (ANOVA, p > 0.05).

**Figure 5:**
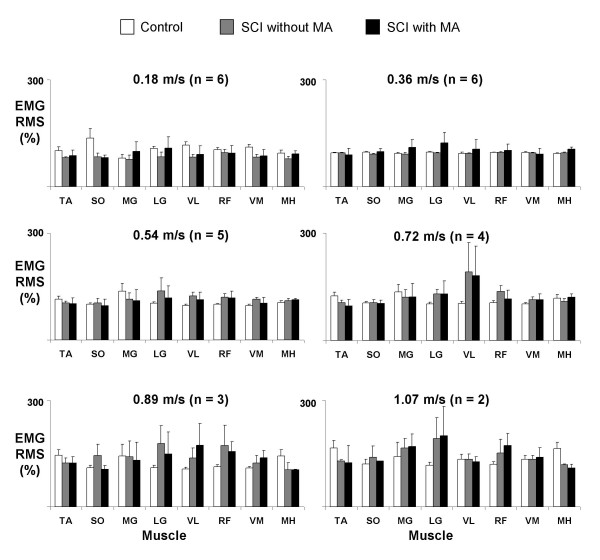
**Swing phase EMG RMS for subjects with spinal cord injury walking with and without manual assistance and control subjects at six different speeds**. Averaged normalized muscle activation amplitudes for tibialis anterior (TA), soleus (SO), medial gastrocnemius (MG), lateral gastrocnemius (LG), vastus medialis (VM), vastus lateralis (VL), rectus femoris (RF), and medial hamstring (MH) for the specified number of subjects with spinal cord injury and 6 control subjects. Bars indicate mean ± standard error. There were no significant differences in muscle activation amplitudes when walking with or without manual assistance (ANOVA, p > 0.05).

Muscle activation amplitudes in subjects with spinal cord injury walking with manual assistance were very similar to amplitudes during walking without manual assistance (Figures 4 and 5). There were no significant differences in normalized EMG RMS between the two conditions for any muscles (ANOVA, p > 0.05), except VM during stance (ANOVA, p = 0.02). Power analyses comparing the differences in means and the least significant values showed that there was a 95% chance that there was no difference in EMG RMS between the two conditions in the SO and VL during the stance phase and MH during the swing phase.

There were increases in muscle activation amplitudes of subjects with spinal cord injury with speed. Stance EMG RMS increased from slowest to fastest speeds for all experimental conditions in soleus (96%), medial gastrocnemius (120%), vastus lateralis (44%), rectus femoris (48%), and vastus medialis (61%) (all p < 0.01) (Figure 4). Swing EMG RMS increased in soleus (61%), medial gastrocnemius (33%), vastus medialis (61%), and vastus lateralis (49%) (all p < 0.04) (Figure 5). The remaining muscles did not have significant increases in EMG RMS (p > 0.05).

The shape of muscle activation patterns in subjects with spinal cord injury tended to become less similar to controls at faster speeds, especially when walking without manual assistance. When comparing the without manual assistance condition to controls, R-values became significantly less from the slowest to the fastest speed in TA (0.85 to 0.83), SO (0.87 to 0.80), MG (0.84 to 0.74), LG (0.85 to 0.74), VM (0.94 to 0.90), and VL (0.94 to 0.90) (ANOVA, p < 0.05). The phase shift also became larger with increasing speed in LG (5 to -26) (p < 0.05). When comparing the manual assistance condition to controls, only the TA had a significantly lower R-value with increasing speed (0.87 to 0.83) (ANOVA, p < 0.05).

### Kinematics

Kinematic profiles in subjects with spinal cord injury walking with manual assistance were very similar to profiles while walking without manual assistance (Figures 1, 2, and 3). Cross-correlation analyses between with and without manual assistance produced correlation values greater than 0.77 and phase lags less than 3% (ANOVA, p < 0.05) (Table 2). There were small differences in range of motion between conditions (Table 3). During swing, knee joint excursion was ~5 degrees greater with manual assistance (ANOVA, p < 0.05). During stance, hip and ankle joint excursion were both ~3 degrees lower with manual assistance (ANOVA, p < 0.05).

**Table 3:** Joint excursions in subjects with spinal cord injury. Average joint excursion for all subjects with spinal cord injury at all possible speeds while walking with or without manual assistance. Data were averaged separately for the stance and swing phase.

**Joint**	**Without Manual Assistance (°)**	**With Manual Assistance (°)**
Ankle		
*Stance*	18.8	15.8*
*Swing*	13.5	14.7
		
Knee		
*Stance*	27.9	28.9
*Swing*	36.1	41.4*
		
Hip		
*Stance*	22.3	19.3*
*Swing*	23.7	22.5

*Indicates significantly different than without manual assistance condition (p < 0.05)

There were differences in the results of the cross-correlation analyses when we compared the shape and timing of kinematic profiles of spinal cord injury subjects walking with and without manual assistance to control subject data. There was a higher R-value and smaller time shift at the knee joint in the comparison of walking with manual assistance to control data than in the comparison of walking without manual assistance to control data (R, ANOVA p = 0.003; time shift, ANOVA p = 0.011) (Table 2). Power analyses showed that the difference in means of the R-value for the ankle joint profile was greater than the calculated least significant value. This indicates that there is a 95% chance that there actually is no difference in R-value between the two conditions in this joint [37].

Range of motion of the joints increased with increasing speed in the subjects with spinal cord injury. At faster speeds, ankle range of motion over the whole gait cycle increased by 63% (ANOVA, p = 0.003). Hip range of motion increased with increasing speed during the stance phase (67%) and swing phase (64%) (ANOVA, p < 0.001).

### Kinematic Variability

**Figure 6:**
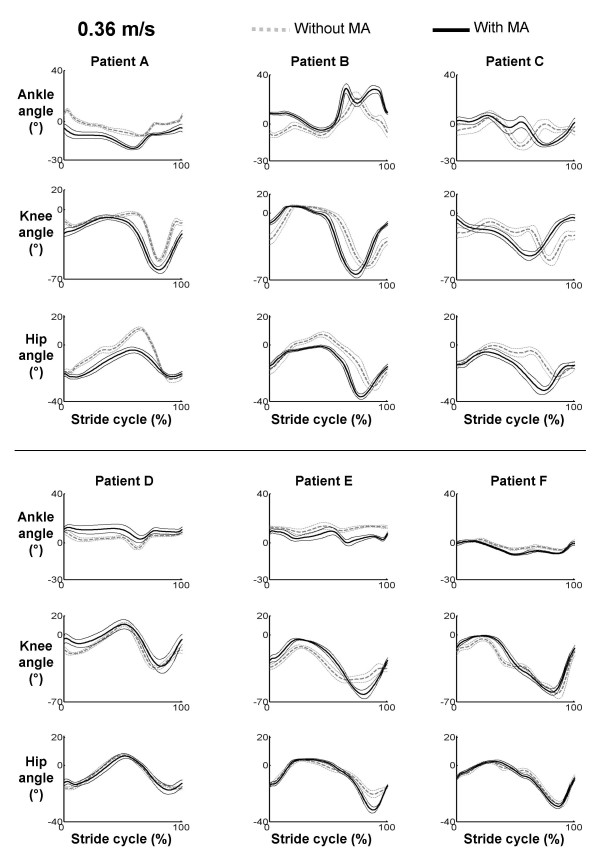
**Kinematic variability in subjects with spinal cord injury**. Figures show joint angle data (heavy line) ± 1 standard deviation (thin lines) for the six different subjects with spinal cord injury walking at 0.36 m/s. Variability increases in some subjects and decreases in others when given manual assistance. Only the ankle joint showed significantly lower variability with subjects were walking with manual assistance.

Variability was less at the ankle joint when subjects with spinal cord injury were given manual assistance (CV = 0.46 without manual assistance, CV = 0.34 with manual assistance, ANOVA, p = 0.03). There were no clear differences in kinematic variability between the with and without manual assistance conditions at the knee or hip (ANOVA, p > 0.05). Figure 6 shows mean joint angles ± 1 SD for all six subjects with spinal cord injury during walking at 0.36 m/s both with and without manual assistance.

## Discussion

The purpose of this study was to determine how manual assistance affected lower limb electromyographic activity and joint kinematics in higher-level subjects with incomplete spinal cord injury during body weight supported treadmill training. We found that muscle activation amplitudes and patterns generally did not change when subjects with spinal cord injury were given manual assistance. Although we expected altered joint excursions with manual assistance, only small changes occurred. There was a small increase in knee joint excursion with manual assistance during swing phase of gait, but this was accompanied by small decreases in hip and ankle range of motion during stance phase. These changes in the joint range of motion excursions were likely due to the facilitation provided by the trainers during manual assistance. Variability of the kinematic profile at the ankle joint decreased when subjects with spinal cord injury were given manual assistance. We also found significant increases in EMG amplitudes and joint excursions with higher walking speeds. The shape of muscle activation patterns in subjects with spinal cord injury also tended to become less similar to controls at faster speeds, especially when walking without manual assistance.

We observed some differences between EMG profiles of control subjects and SCI subjects (Figures 1, 2, and 3). Interpretation of EMG voltages across subjects is generally limited for reasons such as skin impedance, subcutaneous fat thickness, muscle morphology, and electrode placement [38]. Despite this, it is still worthwhile to note some general differences in EMG voltages between control subjects and subjects with spinal cord injury.

The subjects with spinal cord injury adapted to higher speeds differently than the control subjects. At the slowest speed, EMG voltages in the thigh muscles and TA were generally greater in subjects with spinal cord injury than in control subjects (Figure 1). Plantar flexor activation amplitudes were comparable between control subjects and subjects with spinal cord injury at the slowest speed. With faster walking speeds, electromyographic activity in the thigh muscles and TA increased in subjects with spinal cord injury but remained about the same in control subjects (Figure 2 and 3). The most noticeable EMG amplitude difference with speed between SCI and control subjects was in the plantar flexors. Plantar flexor activation greatly increased in control subjects at faster speeds, but there was only a small increase in subjects with spinal cord injury.

There were concurrent changes in kinematics with increasing speed. Ankle plantar flexion increased at terminal stance phase with higher speed in control subjects, but there was less of an increase in this joint angle with speed in the subjects with spinal cord injury. Full knee extension was not achieved by subjects with SCI, and they also tended to be more flexed at the hip than control subjects throughout the gait cycle. These differences in EMG activity and kinematics between control subjects and subjects with spinal cord injury suggest that there are inherent differences in strategies for walking. Because subjects with spinal cord injury have motor deficits, spasticity, and sensory impairments, they must use different patterns of muscle activation and kinematics to accomplish the same functional movements [39].

The difference in adaptation to walking at faster speeds by the control subjects and subjects with spinal cord injury is of importance. The control subjects increased ankle plantar flexor muscle activity at terminal stance to increase their walking speed (Figure 3). The subjects with spinal cord injury lacked this increase in plantar flexor EMG activity. Normally, the ankle joint contributes more mechanical work during walking than the hip or knee [40]. Instead, it appeared that the subjects with spinal cord injury compensated for the lack of ankle power by increasing muscle activity in the hip flexors. This may explain the high net cost of gait in individuals with spinal cord injury [41]. In addition, the inadequacy of ankle push off in terminal stance may prevent patients with spinal cord injury from achieving higher walking speeds [42]. This suggests that providing powered assistance at the ankle joint may be very important when designing robotic devices for rehabilitation [17].

Our findings suggest that manual assistance may help to keep muscle activation patterns more similar to the pattern of control subjects during faster walking speeds. The shape of muscle activation patterns in the subjects with spinal cord injury became less similar to the control patterns at faster speeds, especially when walking without assistance. This is in agreement with previous research that showed walking at fast speeds may be an important part of gait rehabilitation programs in persons with spinal cord injury. Beres-Jones et al. found that faster stepping speeds increase afferent input and efferent activity during walking in individuals with spinal cord injury [28]. Other studies indicated that step training at faster treadmill speeds is more effective at increasing over ground walking speed than step training at slower treadmill speeds in patients with stroke [43,44]. Manual assistance may be beneficial because it allows persons with spinal cord injury to more safely achieve higher walking speeds. Half the subjects with spinal cord injury in this study could walk at faster speeds with manual assistance than without (Table 1).

There are potential limitations to this study. One limitation to this study was the small number of subjects we tested. The small number of subjects is not a major factor in our outcomes. We found significant differences in several variables. For many of the variables we did not find significant differences between conditions (SO and VL EMG amplitudes during the stance phase, MH EMG amplitude during the swing phase, R-values for TA, SO, LG, VM, VL, and ankle joint profiles, and the time shift for SO EMG profile), power analyses showed that testing more subjects would not likely change the results. The least significant value comparisons demonstrated that there was less than a 5% chance of not detecting a difference between conditions when there actually was a difference [37]. Another variable of this study to consider is the ability of the trainers to administer manual assistance. EMG activity and kinematics could vary depending on the ability and experience of the trainers, and how much assistance the trainers give the subjects. In our case, the trainers were under the direct supervision of someone who was trained at a leading center in body weight supported treadmill training (UCLA Department of Neurology). Manual assistance should only provide enough assistance to facilitate normative walking kinematics and not completely overpower the efforts of the patient [45]. Therefore, it is likely more assistance was needed and given at higher walking speeds than at slower speeds. When measurement devices are available to quantify the amount of assistance given without altering the manner in which the assistance should be given, this variable may be included in the statistical analysis. Lastly, subjects with spinal cord injury may adapt to walking on the treadmill with manual assistance over time, which may result in different muscle activation patterns and amplitudes [46]. This is likely to happen if their walking ability improves with training, as it has been shown in previous studies [1-6]. A training study will be necessary to determine the effects of long-term motor adaptations.

Other future studies should involve testing subjects with different levels of impairment or with different neurological injuries since body weight supported treadmill training is used as treatment for a wide range of patients. All of our subjects were classified on the ASIA Impairment Scale as C or D and most of them were community ambulators. This was a necessary part of the study because the design required that the subjects have some walking ability in order to compare walking with and without manual assistance. However, results may be different for subjects with spinal cord injury that have more or less functional impairments than the ones in our study. Patients with neurological conditions other than spinal cord injury, such as stroke, Parkinson's Disease, or cerebral palsy, should also be tested.

## Conclusion

We predicted that EMG activity and joint kinematics would change with manual assistance. The overall result, however, is that EMG amplitudes change little with manual assistance for relatively higher functioning spinal cord injury subjects. There were small but significant differences in joint range of motion with manual assistance. Providing manual assistance is not a detrimental part of body weight supported treadmill training and it allows subjects with spinal cord injury walk at faster speeds than they could without assistance. In addition, manual assistance helps to keep the muscle activation patterns more similar to control data when walking at higher speeds.

## Competing interests

The author(s) declare that they have no competing interests.

## Authors' contributions

AD recruited subjects, managed all data collections, completed all data analysis and drafted the manuscript. GSS recruited subjects, assisted with data collections and edited the manuscript. DPF conceived the study, provided expert guidance on experimental design, assisted with data collections and edited the manuscript. All authors read and approved the final manuscript. This work was supported by the Christopher Reeve Paralysis Foundation grant FAC2-0101 to DPF.

## Supplementary Material

Additional file 1 Spinal cord injury subject walking with manual assistance. This is video of a spinal cord injury subject walking with manual assistance at 0.54 m/s. Click here for file

Additional file 2 Spinal cord injury subject walking without manual assistance. This is video of the same spinal cord injury subject walking without manual assistance at 0.54 m/s. Click here for file
